# Supplementary material for: Polyketide synthase gene clusters in the ericoid mycorrhizal fungus Oidiodendron maius: genome-wide prediction, expression profiling, and functional characterization of knockout mutants
Source: Front Plant Sci. 2026 Sep 16;17:1924430. doi: 10.3389/fpls.2026.1924430 (PMC13623903; doi:10.3389/fpls.2026.1924430)
Supplement: Supplementary file 1 [file SupplementaryFile1.zip › Supplementary Materials/Supplementary Folder 1.zip!/Oidma1_AssemblyScaffolds_Repeatmasked/knownclusterblast/cluster48/jgi.p_Oidma1_60852_mibig_hits.html]

| MIBiG Protein | Description | MIBiG Cluster | MiBiG Product | % ID | % Coverage | BLAST Score | E-value |
| --- | --- | --- | --- | --- | --- | --- | --- |
| AIS24861.1 | Dst17 | BGC0001147 | Distamycin\_biosynthetic\_gene\_cluster | 38 | 95.2247191011 | 211 | 1.5e-54 |
| CAE02620.1 | Yx01\_protein | BGC0000433 | Surfactin\_biosynthetic\_gene\_cluster | 32 | 108.707865169 | 180 | 2.8e-45 |
| BAE95593.1 | putative\_dehydrogenase | BGC0000703 | Kanamycin\_biosynthetic\_gene\_cluster | 25 | 85.1123595506 | 81 | 3e-15 |
| CAF60509.1 | putative\_dehydrogenase | BGC0000704 | Kanamycin\_biosynthetic\_gene\_cluster | 25 | 85.1123595506 | 81 | 3e-15 |
